# Magnesium Deficiency Reduced the Yield and Seed Germination in Wax Gourd by Affecting the Carbohydrate Translocation

**DOI:** 10.3389/fpls.2020.00797

**Published:** 2020-06-11

**Authors:** Baige Zhang, Ismail Cakmak, Jianchun Feng, Chaoran Yu, Xiao Chen, Dasen Xie, Liangquan Wu, Zhao Song, Jian Cao, Yuzhi He

**Affiliations:** ^1^Key Laboratory for New Technology Research of Vegetable, Vegetable Research Institute, Guangdong Academy of Agricultural Sciences, Guangzhou, China; ^2^Faculty of Engineering and Natural Sciences, Sabanci University, Istanbul, Turkey; ^3^International Magnesium Institute, Fujian Agriculture and Forestry University, Fuzhou, China

**Keywords:** magnesium deficiency, wax gourd, carbohydrate, seed germination, fruit yield

## Abstract

Magnesium (Mg) is a particular mineral nutrient greatly affecting the size and activity of sink organs. Wax gourd crop with its fruits having fresh weight up to 20–50 kg per single fruit serves as an excellent experimental plant species for better understanding the role of varied Mg nutrition in sink strength and yield formation. This study aimed to investigate the effects of Mg deficiency on fruit yield and seed vigor in wax gourd grown under field conditions. Plants were grown under field conditions until maturity with increasing soil Mg applications. At the beginning of fruit formation, leaves were used to analyze concentrations of sucrose, starch and Mg as well as phloem export of sucrose. At maturity, fruit yield was determined and the seeds collected were used in germination studies and starch analysis. Low Mg supply resulted in a significant impairment in fruit fresh yield, which was closely associated with higher accumulation of starch and sucrose in source leaves and lower amount of sucrose in phloem exudate. Seeds obtained from Mg deficiency plants exhibited lower amount of starch and substantial reduction in both germination capacity and seedling establishment when compared to the seeds from the Mg adequate plants. Our study revealed that magnesium deficiency significantly diminished fruit yield of field-grown wax gourd, most probably by limiting the carbohydrate transport from source organs to developing fruit. Ensuring sufficient Mg supply to plant species with high sink size such as wax gourd, during the reproductive growth stage, is a critical factor for achieving higher fruit yield formation and also better vigor of next-generation seeds.

## Introduction

Magnesium (Mg) has diverse critical physiological functions in plant cells. The well-documented function of Mg is related to its particular role in development and formation of sink organs such as roots and seeds (Cakmak and Kirkby, 2008; Verbruggen and Hermans, 2013; Ceylan et al., 2016). Magnesium deficiency most commonly occurs in regions where highly weathered and acidic soils are widespread and intensive cropping system with high Mg removal from soils takes place (Gransee and Fuhrs, 2013; Wang et al., 2019). It has been also reported that Mg concentrations in cereals crops showed a clear decline over the past 60 years, most probably due to dilution of Mg associated with marked increases in grain yield as well as due to imbalanced mineral fertilization without considering crop demand for Mg (Guo et al., 2016)

The most typical symptoms of Mg deficiency in crop plants is the development of interveinal leaf chlorosis on older leaves (Marschner, 2012; Senbayram et al., 2015). Leaves became interveinal yellowish under low Mg supply due to both photooxidative degradation of chlorophyll and inhibition of its biosynthesis (Cakmak and Kirkby, 2008; Traenkner et al., 2018). Published reports show that accumulation of photoassimilates in leaves is a very early response of plants to Mg-deficiency stress and it happens before the development of leaf chlorosis (Cakmak et al., 1994a, b; Hermans et al., 2005). Kobayashi and Tanoi (2015) showed an over-accumulation of sucrose and starch in source leaves several days before visual leaf symptoms appeared. The reported increases in accumulation of carbohydrates in source leaves is attributed to a fundamental role of Mg plays in phloem export of photosynthates (Cakmak et al., 1994a; Hermans and Verbruggen, 2005; Farhat et al., 2016). Low Mg nutrition diminishes phloem loading of sucrose, probably due to low availability of Mg-ATP, which is required for the activity of H^+^-ATPase activity of sieve tube plasma membranes to maintain phloem loading of sucrose (Cakmak and Kirkby, 2008; Kobayashi and Tanoi, 2015). Mg is known to be needed both for biosynthesis and function of ATP (Cakmak and Kirkby, 2008; Igamberdiev and Kleczkowski, 2015). Main binding form of ATP in cellular systems is Mg-ATP which serves as a common substrate for the plasma membrane ATP’ases for the transport process such as uptake of sucrose into phloem channel (Gout et al., 2014). Previously, Cakmak et al. (1994a) showed that phloem export of sucrose is very sensitive to low Mg supply and a resupply of Mg to Mg-deficient plants for only 12h significantly regenerated phloem transport of sucrose. This rapid positive effect of Mg on phloem transport might be related to increases in the pool of Mg-ATP and/or increases in the sink activity. A further explanation for the reduced phloem transport of photoassimilates under low Mg supply might be also related to the decrease in sink size resulted from Mg deficiency stress in plants (Mengutay et al., 2013).

The well-known decreases in root growth of Mg-deficient plants are associated closely with low amounts of sugars in roots (Cakmak and Kirkby, 2008; Farhat et al., 2016; Traenkner et al., 2018). According to the results of Guo et al. (2015) in Arabidopsis plants, root growth is severely affected by marginal Mg supply; but, root hair formation was stimulated. In soybean plants, the weight and size of nodules are strongly reduced under low Mg, and these decreases were found to be in close association with the corresponding decreases in starch and sucrose concentrations of roots and nodules (Peng et al., 2018). Similarly, also seed size and seed weight of wheat plants were markedly reduced under Mg deficiency, and a foliar spray of Mg to Mg-deficient plants reversed the detrimental effects of low Mg on seed size (Ceylan et al., 2016). Ceylan et al. (2016) also showed that the level of starch in seeds reduced by Mg deficiency while there was a high starch accumulation in leaves. Very recently, in potato plants, Koch et al. (2019) showed similar impairments in growth of roots and tubers under low Mg supply which was also linked to the impaired delivery of carbohydrates from shoot into roots, especially in case of higher potassium (K) applications. Due to antagonistic interactions of high K with root uptake and root-to-shoot translocation of Mg, plants under low Mg supply are very sensitive to high applications of K and develop Mg deficiency stress (Senbayram et al., 2015). These results clearly demonstrate that Mg deficiency has high detrimental impact on growth and size of sink organs such as roots, seeds and nodules by impairing delivery of carbohydrates from the source leaves.

However, all the experiments mentioned and discussed above have been realized under controlled growth chamber or greenhouse conditions. To the best of our knowledge, field experiments analyzing sugar transport from source into sink organs and changes in development of sink organs under different Mg treatments are not known. It has been already reported that the plant response to varied Mg nutrition in terms of dry matter allocation between shoots and roots is highly affected by the experimental cultivation conditions (Guo et al., 2016; Hauer-Jakli and Traenkner, 2019; Wang et al., 2019). There is also a growing debate on relevance and constraints of the pot experiments conducted under greenhouse or growth chamber conditions. Transferability of the experimental results from the pot experiments to the real-world conditions represents an important discussion point because source-sink relationships, biomass allocation, root response to the studied treatment and leaf photosynthetic rate etc. are greatly affected from pot size and greenhouse growth conditions used (Poorter et al., 2012, 2016; Rebetzke et al., 2014; Aragao et al., 2020). It is, therefore, of importance to investigate how Mg deficiency affects sink strength, sugar accumulation in source and sink organs as well as phloem export of sucrose in a field-grown plant such as wax gourd having a substantial sink size.

Wax gourd [*Benincasa hispida* (Thunb.) Cogn., Tiezhu cv] tis known as a crop with a strong sink strength and produces normally over 20 kg (Xie et al., 2019) or in some cases up to 50 kg (Dhillon et al., 2016) fresh weight per single fruit. Wax gourd is widely cultivated in tropical and subtropical regions of the world, usually consumed as vegetable and contains high level of valuable nutrients and even used in the traditional medicine (Marr et al., 2007; Ghosh and Baghel, 2011; Nurul et al., 2011). Our field observations show clearly that Mg deficiency occurs commonly in wax gourd fields, and growers are, however, not well aware of this problem. As the case with other crop plants (Romheld and Kirkby, 2010) leaf Mg deficiency symptoms are often mistakenly ascribed to K deficiency. Thus, the findings of this paper may have also possible practical implications. In the present study, our aim was to study effects of varied Mg nutrition on growth and yield formation as well as phloem export and concentrations of sucrose in source leaves of wax gourd plants grown under field conditions. Attention has been also paid to seed germination, seed starch and seedling development under varied Mg treatments.

## Materials and Methods

### Site Description

**FIGURE 1:**
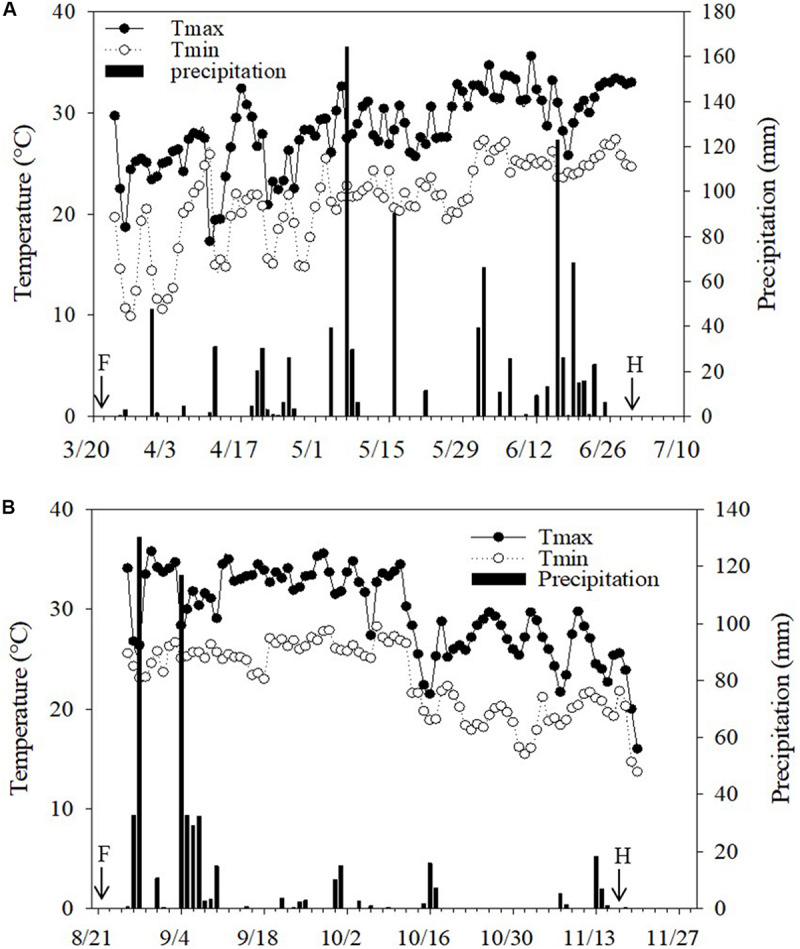
Daily precipitation and temperature during wax gourd growing seasons at the field sites in **(A)** 2017 spring and **(B)** 2017 autumn. F: Application date for Mg fertilizers. The transfer of the seedlings was realized right after fertilization. H: Harvesting time of fruits. Total precipitation during the experiment was 960 mm in 2017 spring and 504 mm in 2017 autumn.

Two rotational field experiments were carried out in Guangzhou (23°08′N,113°16′E), China, from March to December 2017. The climate in the region is a subtropical marine monsoon climate with an average temperature of 23°C and an annual precipitation of 1800 mm. The daily temperature and precipitation during the growing season of wax gourd in experimental fields are given in Figure 1.

Soils of the field experiments were lateritic soils, and the soil samples were taken from the topsoil (30 cm depth) prior to sowing for measurement of several chemical and physical properties. The soil was sandy loam, had a pH value of 6.1 and contained 21.4 g kg^–1^ organic matter measured by using standard Walkley-Black method. The amount of exchangeable Mg of the soils was 104 mg kg^–1^ and measured by using ammonium acetate (NH_4_OAc) method described by Rice and Kamprath (1968). The Olsen-P and NH_4_OAc-exctractable K of the soils were 6.1 mg kg^–1^ and 190 mg kg^–1^ which were determined according to Lu D. et al. (2017) and Lu et al. (2019), respectively.

### Establishment of the Field Experiments

Seeds of *Benincasa hispida* cv. Tiezhu 2 were soaked in deionized water for 8 h, wrapped in damp paper, and then kept in a plastic bag for germination. Germinated seeds were then transplanted to the field at the three-true-leaf stage. The experiment was conducted using a randomized block design with four replicates after transplantation. Each plot was 45 m^2^, being 30 m in length and 1.5 m in width, and included 6 rows. The row distance was 1.2 m. Wax gourd plants were grown until maturity with five soil Mg applications, namely 0, 30, 60, 90, and 120 kg Mg ha^–1^ in the form of synthetic MgSO_4_ ⋅ H_2_O. Other nutrients were uniformly applied to all plots including 450 kg N ha^–1^, 225 kg P_2_O_5_ ha^–1^, 375 kg K_2_O ha^–1^ in the form of urea, superphosphate and potassium sulfate, respectively. Of the Mg treatments mentioned, 0 kg ha^–1^ Mg application and 90 kg ha^–1^ Mg application were considered as control treatment (−Mg; Mg deficiency treatment) and the adequate Mg treatment (+Mg). The middle rows of the plots were used for the plant sampling and harvesting.

When plants were matured 90 days after transplanting, the fruits were harvested and weighted for determination of fruit yield, and its circumference and flesh firmness were detected immediately. The seeds collected from the fruits were washed in dH_2_O, dried at room temperature, and stored for germination tests. The plant leaf samples were taken from four uniform plants within each plot. Plant samples were washed in dH_2_O. All plant samples were put in paper bags, dried 105°C for 30 min and then at 80°C for 3 days, and then weighed at room temperature. Dried plant samples were ground to fine powders for future analysis. The fruit yield and yield components, and biomass distribution of wax gourd were measured in two season’s experiments.

### Measurements of Leaf Mg, Starch, and Sucrose

The first leaf above fruit were used for analysis of Mg, starch and sucrose as well as for collection of phloem exudates. For the determination of Mg, dried plant samples were ground to fine powders, and approximately 0.2 g from each ground sample was acid-digested by using 2 ml of 30% H_2_O_2_ and 5 ml of 65% HNO_3_ in a closed-vessel microwave system. After the digestion, Mg existing in the digests was analyzed by inductively coupled plasma-mass spectrometry (ICP-AES, model IRIS, TJA, United States).

The analysis of sucrose and starch was performed by using a spectrophotometric method after extraction of the corresponding leaf or seed samples and extracted three times in 5 ml 80% (v/v) ethanol for 30 min at 20°C. The three supernatants were combined and diluted to 25 ml with 80% ethanol, mixed and stored at −20°C. Sucrose was assayed on the re-suspended supernatant by considering the method described by Hendrix (1993). Starch was extracted by incubating the residue left after ethanolic extraction with HClO_4_ overnight according to Seifter et al. (1950) The starch concentration was then analyzed by using spectrophotometer (UV-1100, Mapada Instruments Ltd., Shanghai, China) at 620 nm, and glucose was used as the standard.

### Collection of Phloem Exudate

The collection of phloem exudates was carried out according to Cakmak et al. (1994a) by using the first leaf above fruit. Immediately after excision of the leaves from the shoots, the leaf petioles were recut under distilled water and placed in a glass vial containing 5 ml 20 mol m^–3^ EDTA solution (pH 6.0). About 0.5 cm of the petiole was immersed in solution. During the collection of phloem exudate, samples were incubated in an air-tight plexiglass chamber containing water to maintain high humidity. Following 6 h incubation in darkness, collection of exudates was terminated and the exudate solutions were used for measurement of sucrose spectrophotometrically.

### Flesh Firmness

**FIGURE 2:**
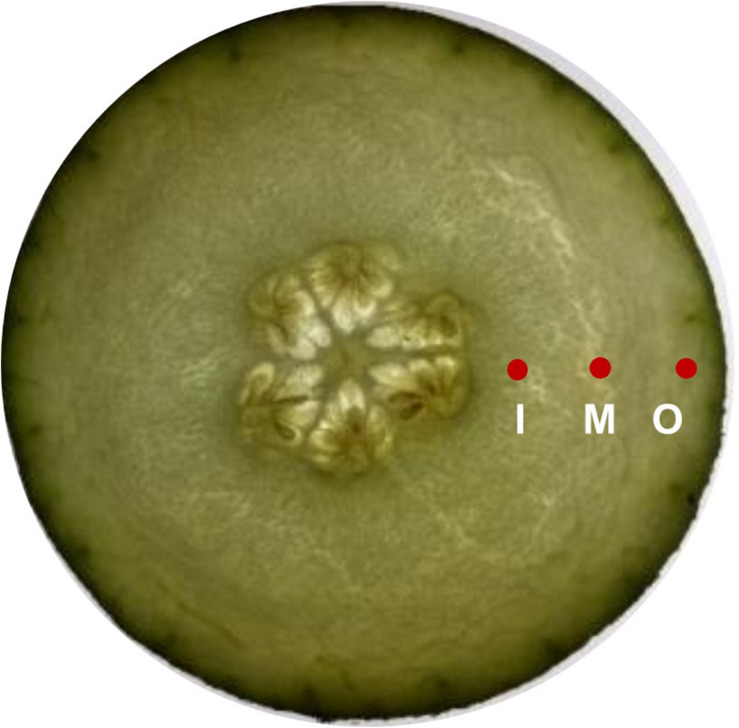
Sketch of firmness measurement positions. I, Inner; M, middle; O, Outer position.

Fruit firmness was measured using FHM-5 durometer (Takemura Electric Works Co., Ltd., Tokyo, Japan) with a conical probe (tip diameter 12 mm, height 10 mm). External firmness was determined by four readings taken on each fruit at 1 cm distance to peel, while the internal pulp firmness was measured at four locations from about 1 cm^2^ distance to the fruit core and the durometer was employed to 1 cm depth. The middle was defined as position between inner and outer. The measurement positions are given in Figure 2.

### Germination Tests

Germination tests were realized under greenhouse conditions by using filter paper and perlite media. Seeds produced by the experimental plants grown with varying Mg supply were collected from fruits and used in the germination tests. Seeds were first soaked in deionized water for 8 h and then placed between sheets of wet filter papers. This was a 3-replicate test, and 20 seeds were used for each replicate. The germination rate was calculated at 3rd day. Thereafter, the germinated seeds were transplanted into perlite medium and the dry weights of all seedlings were measured when the seedlings were 15 days old in the perlite medium.

### Statistical Analysis

Statistical analysis of the data was conducted by using JMP (12.0.1) (SAS Institute Inc., Cary, NC, United States). The significance of treatment effects was evaluated by analysis of variance (ANOVA). Then, Tukey’s honestly significant difference (HSD) test (*p* < 0.05) was used as a *post hoc* test to determine significant differences between means. Additional information on statistic (i.e., replications) are given in the legend of the tables and figures.

## Results

The air temperature was generally similar in both cropping seasons, while natural rainfall greatly varied (Figure 1). Total rainfall during cropping seasons was more (960 mm) and uniformly distributed in the spring season while in case of autumn season, the total rainfall was 504 mm which was mostly concentrated in the beginning of the season (Figure 1). Most probably, the mentioned uneven distribution of rainfall was the major reason for the lower fruit yield in autumn than the spring season (Table 1).

**TABLE 1:** Changes in fruit yield of wax gourd (*Benincasa hispida* cv. Tiezhu 2) plants depending on increasing Mg supply in form of MgSO_4_ ⋅ H_2_O in two seasons of 2017 under field condition*.

**Mg Supply (kg ha^–1^)**	**Spring season (t ha^–1^)**	**Autumn season (t ha^–1^)**
0	113.7 e	61.0 c
30	114.9 d	69.2 b
60	121.7 c	74.2 a
90	129.5 a	76.0 a
120	124.0 b	61.6 c

*Values are means of four independent replicates. Different letters indicate significant differences between means according to one-way ANOVA and Tukey’s HSD test (p ≤ 0.05).

**FIGURE 3:**
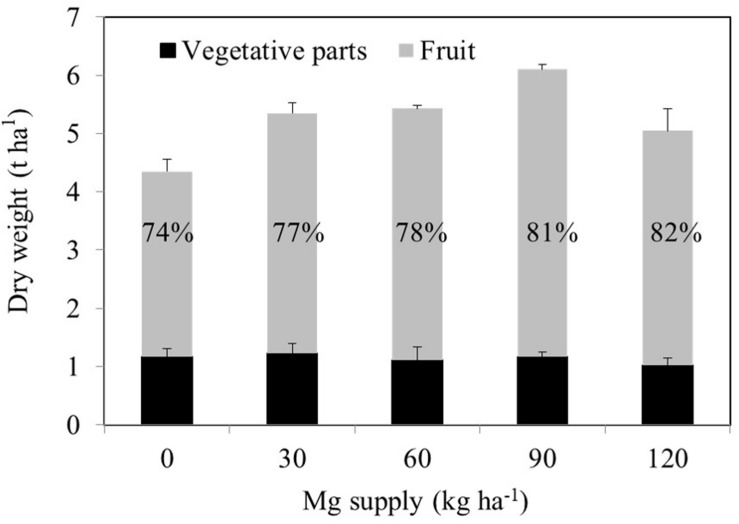
Dry weights of vegetative tissues of 120 days old mature wax gourd (*Benincasa hispida* cv. Tiezhu 2) plants grown with increasing Mg supplies in form of MgSO_4_ ⋅ H_2_O under field conditions in 2017 spring season. Values are means of four independent replicates. The numbers on the columns show the ratio of the fruit dry weight to total dry weight.

**FIGURE 4:**
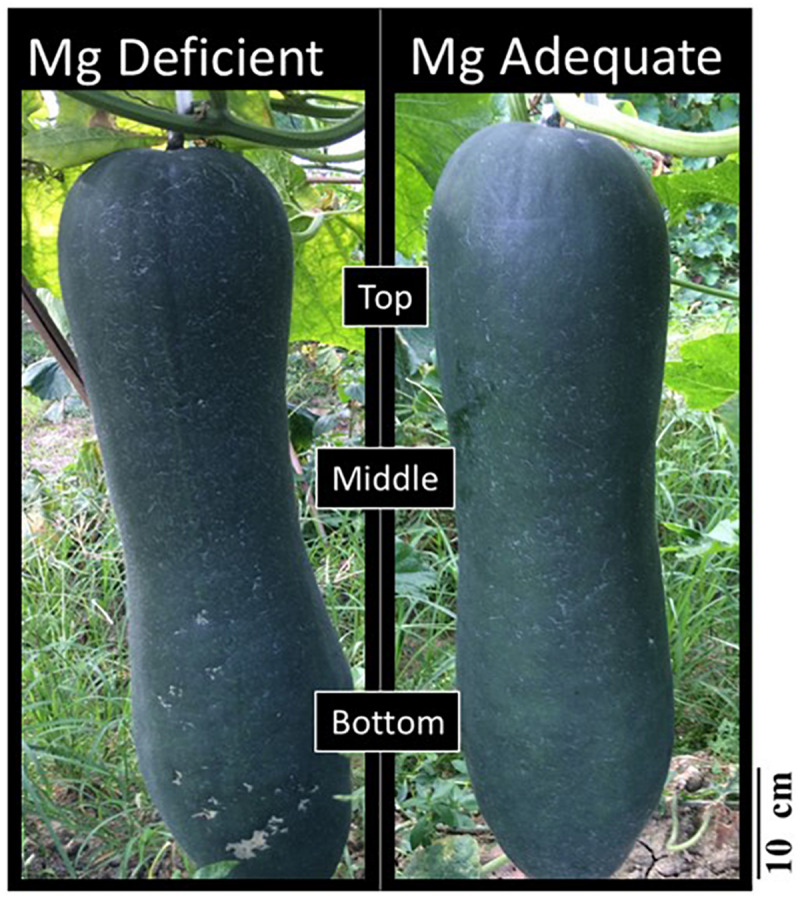
Fruits at maturity obtained from wax gourd plants (*Benincasa hispida* cv. Tiezhu 2) grown with (i) no Mg and (ii) adequate Mg supply (90 kg ha^–1^ MgSO_4_ ⋅ H_2_O) under field conditions in 2017 spring season.

A consistent trend in the yield response of plants to increasing Mg fertilization was found in both cropping seasons (Table 1). Enhancement in Mg application up to 90 kg ha^–1^ resulted in a gradual increase in the yield of wax gourd over two consecutive seasons (Table 1). At the application of 120 kg Mg ha^–1^, there was a decline in yield in both growing seasons (Table 1), suggesting that the Mg treatment of 90 kg ha^–1^ could be the suitable Mg application rate under given experimental conditions. In contrast to the fruit yield, Mg applications had a little effect on the dry weights of vegetative part of the plants at maturity (Figure 3). The dry matter (DM) allocation from vegetative parts to fruits was highest at the Mg application rate of 90 kg ha^–1^ (Figure 3). The fruit DW/total DW ratio was 74% at lowest Mg application and 81% with the 90 kg ha^–1^ Mg application (Figure 3). Adequate Mg nutrition exhibited also significant effects on fruit shape (Figure 4 and Table 2). The fruit diameter was almost same at the bottom part of the fruits between low and adequate Mg treatments, while adequate Mg treatment significantly enhanced fruit diameter in the middle and top parts (Table 2 and Figure 4). Fruit flesh firmness was also improved in each part of the fruits by sufficient Mg treatment as shown in Table 2.

**TABLE 2:** Fruit circumference and flesh firmness of wax gourd (*Benincasa hispida* cv. Tiezhu 2) plants grown without (−Mg) and with Mg supplies (+Mg: 90 kg ha^–1^ MgSO_4_ ⋅ H_2_O) under field conditions in spring season of 2017*.

**Mg supply**	**Fruit circumference (cm)**			**Flesh firmness (kg cm^–2^)**		
	**Top**	**Middle**	**Bottom**	**Outer**	**Middle**	**Inner**
− Mg	56.5 b	54.1 b	61.4 b	2.18 b	0.93 b	0.74 b
+ Mg	60.4 a	58.2 a	61.8 ab	2.71 a	1.28 a	0.84 a

*Values are means of four independent replicates, and each replicate included four samples. Different letters indicate significant differences between means according to one-way ANOVA and Tukey’s HSD test (p ≤ 0.05).

**FIGURE 5:**
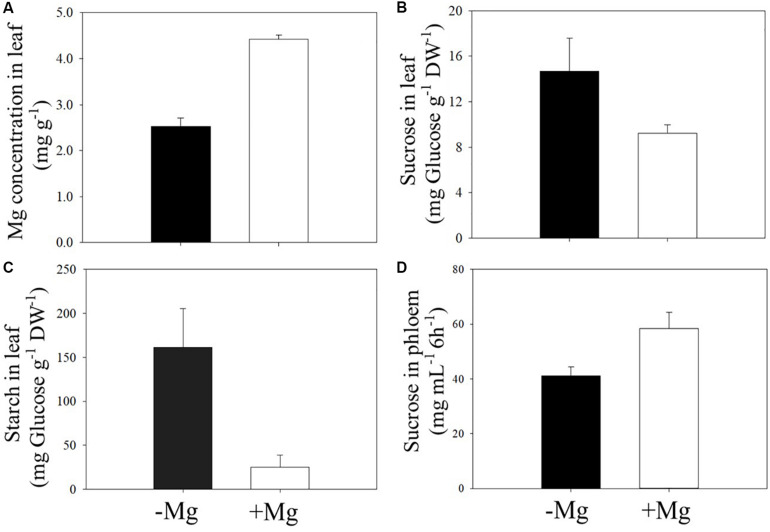
Effect of sufficient (+Mg; 90 kg ha^–1^ MgSO_4_ ⋅ H_2_O) and deficient supply of Mg (−Mg; no supply) on the concentrations of Mg **(A)**, sucrose **(B)**, and starch **(C)** measured in the first leaf above the fruit. Same leaves were also used for measurement of sucrose in phloem exudates **(D)** at beginning of fruit formation stage in 2017 spring season. Vertical bars represent the mean ± SE of four independent replicates.

As expected, deficient supply of Mg reduced leaf Mg concentration (Figure 5A), and this decrease in leaf Mg is associated with higher accumulation of starch and sucrose in Mg-deficient leaves (Figures 5B,C). The accumulation of starch in Mg-deficient leaves was about sixfold higher when compared to the Mg-adequate plants (Figure 5C). In contrast to leaf sucrose concentration, Mg deficiency reduced the concentration of sucrose in phloem exudate collected from the same leaves. The amount of sucrose in the phloem exudates without Mg application was about 30% less than those plants adequately supplied with Mg (Figure 5D).

**FIGURE 6:**
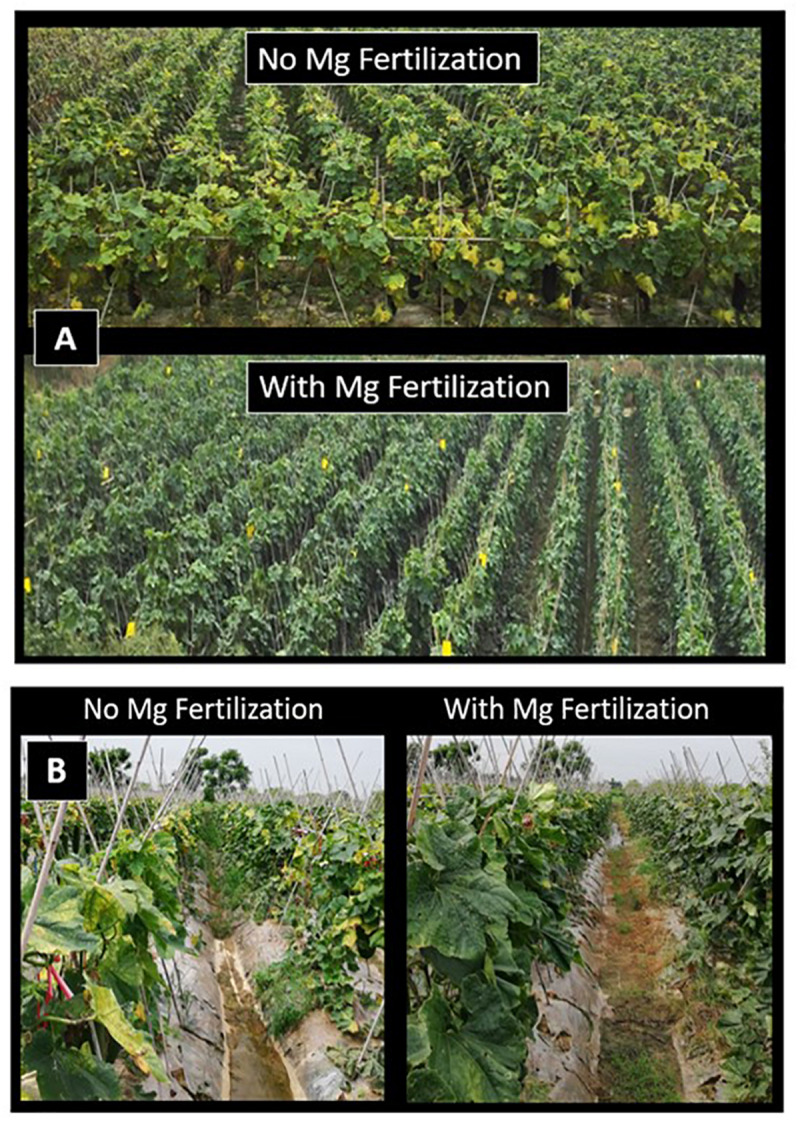
Wax gourd plants grown under farmers’ field conditions with (i) no Mg and (ii) adequate Mg treatments (90 kg ha^–1^ MgSO_4_**⋅** H_2_O) at Sanshui district in 2017 **(A)** and Datang district in 2018 **(B)**, Foshan city, Guangdong province, China.

Very distinct Mg deficiency symptoms (i.e., intercostal chlorosis) were also observed on leaves of wax gourd plants grown under farmer’s field conditions which received no Mg fertilization (Figure 6). In 2017 and 2018 spring seasons, Mg application promoted fruit yield of wax guard in farmer fields by about 30 and 23%, respectively (Table 3). It is obvious from the pictures that Mg deficiency in farmer fields also clearly reduced chlorophyll concentrations of leaves (Figure 6). It was interesting to note that the interveinal leaf chlorosis was first developed on the leaves above the fruits. Thereafter, older leaves started to develop leaf chlorosis under low Mg supply.

**TABLE 3:** Yield performances of wax gourd (*Benincasa hispida* cv. Tiezhu 2) plants grown without (−Mg) and with Mg supplies (+Mg: 90 kg ha^–1^ MgSO_4_ ⋅ H_2_O) under farmer’s field conditions in 2017 and 2018 spring season*.

**MgSO_4_ fertilization**	**Yield (t ha^–1^)**	
	**2017 spring season**	**2018 spring season**
− Mg	75.0 b	99 b
+ Mg	97.5 a	122 a

*Wax gourd plants were grown in farmer’s fields at Sanshui district in 2017 and Datang district in 2018, Foshan city, Guangdong province without (−Mg) and with Mg supplies (+Mg: 90 kg ha^–1^ MgSO_4_ ⋅ H_2_O). Yield values presented are the mean values of 16 independent samples in each treatment.

**FIGURE 7:**
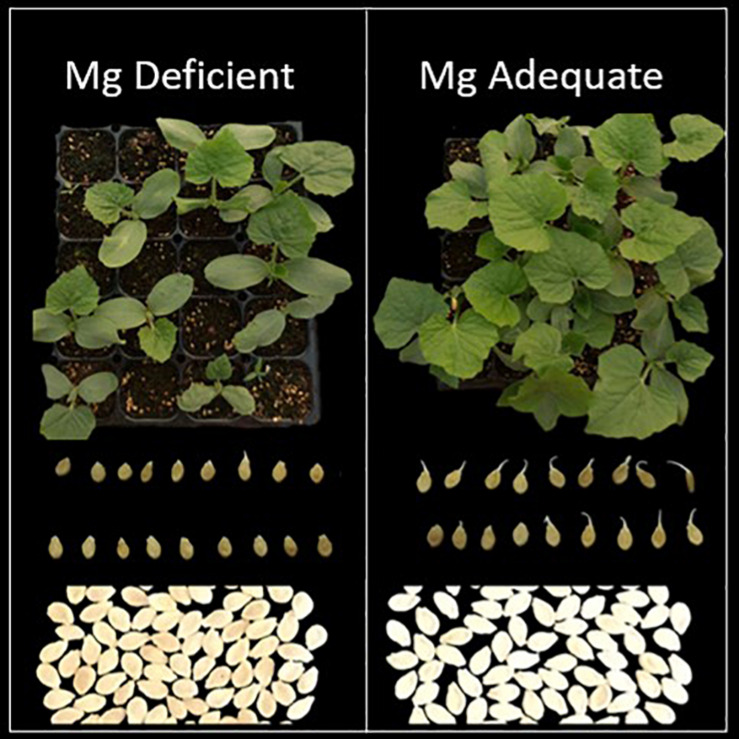
Appearance of seeds and their germination and development. The seeds used in the germination studies were obtained from the wax gourd plants (*Benincasa hispida* cv. Tiezhu 2) grown with (i) no Mg and (ii) adequate Mg supply (90 kg ha^–1^ MgSO_4_ ⋅ H_2_O) under field conditions in 2017 spring season. The germinating seeds were 3 days old and the seedlings were 15 days old.

When compared to the seeds collected from the plants grown with adequate Mg, the seeds from the Mg-deficient plants exhibited thinner shape, slight brownish color and reduced size (Figure 7). The total number of seeds per fruit obtained from the plants without Mg treatment was about 25% less when compared to the seed numbers from the Mg adequate plants (Table 4). Seed germination studies showed that the seeds derived from Mg-sufficient plants were more vigorous and exhibited earlier seed emergence and better seedling establishment and development compared to the seeds collected from Mg deficient plants (Table 4 and Figure 7). There was a particular decrease in germination rate with the seeds derived from the Mg deficient plants. The dry weight of seedlings was also significantly reduced with the seeds from the Mg deficient plants. As expected, Mg-deficient seeds had lower Mg concentrations (Table 4). Seeds were also analyzed for the starch concentrations and total amount of seed starch per fruit (Table 5). Low Mg nutrition reduced the starch concentration by about 33%. Because the seed yield was reduced under Mg deficiency (Table 4), total amount of seed starch was at much greater extend reduced by Mg deficiency (Table 5).

**TABLE 4:** Seed weight per fruit, seed Mg concentration, germination rate, and dry weight of seedlings including shoot and root from seeds of wax gourd (*Benincasa hispida* cv. Tiezhu 2) plants grown under field conditions with no (−Mg), and adequate (+Mg: 90 kg ha^–1^ MgSO_4_ ⋅ H_2_O) Mg under greenhouse conditions in the spring season of 2017*.

**Mg supply**	**Seed yield (g fruit^–1^)**	**Mg concentration (g kg^–1^)**	**Germination rate (%)**	**DW of seedling (g plant^–1^)**
− Mg	65.9 b	1.76 b	20 b	0.19 b
+ Mg	82.3 a	1.97 a	85 a	0.29 a

*Values are means of three independent replicates and 20 seeds were used for each replicate for germination test. Different letters indicate significant differences between means according to one-way ANOVA and Tukey’s HSD test (p ≤ 0.05).

**TABLE 5:** Seed starch concentrations and starch content per fruit of wax gourd (*Benincasa hispida* cv. Tiezhu 2) plants grown under field conditions with no (−Mg), and adequate (+Mg: 90 kg ha**^–^**^1^ MgSO_4_ ⋅ H_2_O) Mg in the spring season of 2017*.

**Mg supply**	**Starch concentration (mg g^–1^)**	**Starch content (g fruit^–1^)**
− Mg	32.5 b	2.14 b
+ Mg	43.1 a	3.55 a

*Values are means of four independent replicates and 30 seeds were used for each replicate for starch test. Different letters indicate significant differences between means according to one-way ANOVA and Tukey’s HSD test (p ≤ 0.05).

## Discussion

The present study clearly demonstrated that Mg deficiency has detrimental impacts on fruit yield of wax gourd plants grown under field conditions. Wax gourd fruit has a particular size and weight (Figure 4). In the present study, the fresh weight of a single fruit under sufficient Mg supply was around 20 kg, and the fruit formation is completed within about 30 days, suggesting a strong demand and fast flow of carbon in the wax gourd fruits during the fruit development. These findings also highlight importance of the phloem loading and transport of photoassimilates in wax gourd plants for the yield formation.

Phloem loading process for sugars is known to be very sensitive to Mg nutritional status of the plants. Among the plant mineral nutrients, Mg together with potassium (K) are particular mineral nutrients in terms of their positive impacts on phloem loading and transport of sucrose (Cakmak, 1994; Cakmak et al., 1994a; Verbruggen and Hermans, 2013; Traenkner et al., 2018). The reductions in fruit yield (Tables 1, 3) and fruit size (Table 2 and Figure 4) are most likely related to reduced transportation of assimilates from leaves in the fruits. It is widely believed that phloem loading process of sucrose takes place with the involvement of a plasma membrane-localized sucrose proton symporter that uses energy derived from ATP hydrolysis (Marschner, 2012; Lemoine et al., 2013). Most probably, the ATP-substrate used in generation of the energy is Mg-ATP (Cakmak and Kirkby, 2008; Igamberdiev and Kleczkowski, 2015). It is known that the activity of most ATPases existing in biological systems depends on availability of Mg-ATP (White and Marschner, 2012) and it has been speculated that lack of available Mg-ATP in phloem loading zones in source organs is most likely the major reason why plants under low Mg supply cannot translocate adequate amount of photoasasimilates from source in the sink organs (Cakmak and Kirkby, 2008; Lemoine et al., 2013; Mengutay et al., 2013; Yang et al., 2019).

In good agreement with the proposed role of Mg in phloem loading and transport of assimilates, phloem export of sucrose in field grown wax gourd was clearly reduced (Figure 5D). Lower amount of starch in seeds of Mg deficient plants despite lower yield also indicate impairments in the translocation of assimilates from leaves in the fruits (Table 5). This decrease in assimilate transport is well associated with the increases in concentration of starch and sucrose in leaves under low Mg supply (Figures 5B,C). Accumulation of starch in Mg-deficient leaves was particularly high (Figure 5C) while the leaf accumulation of sucrose is less pronounced (Figure 5B). Sucrose is known to be the major export form of photoassimilated carbon from source in the sink organs in most of the plant species. However, in some plant species, such as the cucurbitaceae family plants, stachyose represents the major transport form of sugars (approximately 40% of the transported sugars) in phloem as shown in cucumber plants (Hu et al., 2011; Lu J. et al., 2017). An inhibition of phloem transport of stachyose through a cold stress treatment was shown to be associated with high starch accumulation (Lu D. et al., 2017). In the present study, since the sucrose accumulation is less pronounced in Mg deficient leaves compared to starch, it might be speculated that the stachyose is most likely the major form of sugar transport in wax guard which is also a member of the cucurbitaceae family. Further studies are needed to investigate the effect of varied Mg nutrition on phloem export of stachyose in wax gourd plants.

As indicated above, the shape and size of fruits (Table 2 and Figure 4) were significantly reduced by low Mg supply which can be also ascribed to limited transportation of carbohydrates in the fruits. These suggestions are well supported with the differential dry matter allocation between vegetative parts and fruits under low and adequate Mg supplies. As presented in Figure 1 and Table 2, the field-grown wax gourd plants with adequate Mg treatment allocated more dry matter into fruits and showed higher flesh firmness than the plants with no or very low Mg treatments. This result is also in good agreement with the lower levels of starch in seed of Mg deficient plants (Table 5). A loss of flesh firmness is largely associated with the cell wall polysaccharides, including pectin, hemicellulose and cellulose factions (Zhang et al., 2019) which is consistent with fruit size and dry weight. Reduced dry matter allocation in the sink organs such as in the youngest shoot tips (Hermans et al., 2005; Mengutay et al., 2013) roots (Cakmak et al., 1994b; Cakmak and Kirkby, 2008) tubers (Koch et al., 2019), and seeds (Ceylan et al., 2016) have been shown under low Mg supply. In all these studies, experimental plants were grown under controlled growth conditions. In recent years, there is a growing debate on the constraints of the pot experiments conducted under controlled growth conditions regarding the transferability of the experimental results and conclusions to the real-word conditions. Published data show that the source-sink relationships, biomass allocation, root response to the studied treatment and leaf photosynthetic rate etc. are greatly affected from the pot size and growth conditions used (Poorter et al., 2012; Rebetzke et al., 2014; Aragao et al., 2020). The results of the present study clearly show that the effects of varied Mg treatments on accumulation and phloem transport of sugars in plants grown under highly controlled growth conditions also occurs, similarly, in plants grown under field conditions. To our knowledge, this study is the first study that investigated the effects of varied Mg nutrition on the changes in sugar concentrations of the source and sink organs and phloem transport of sugars in plants grown under field conditions.

It was interesting to observe that the typical interveinal chlorosis symptom on leaves was first developed on the leaves above the fruit, and exposed directly to sunlight (Figure 6). These leaves contained particular increases in starch accumulation (up to sixfold compared to Mg-adequate leaves; Figure 5). It is therefore suggested that inhibited assimilate translocation from those leaves in combination with high light exposure resulted in photooxidative damage by inducing generation of reactive oxygen species (ROS). Extensive production of ROS at the expense of impaired photosynthesis due to reduced phloem export of assimilates has been already discussed for Mg deficient plants (Cakmak, 1994; Cakmak and Kirkby, 2008; Kobayashi and Tanoi, 2015; Traenkner et al., 2018). Typically, Mg deficiency symptoms first develop on older leaves. However, if the older leaves are significantly shaded by the upper leaves and even by large fruits as the case with wax gourd plants, development of the Mg deficiency symptoms on older leaves might be delayed. Previously, Marschner and Cakmak (1989) showed that a partial shading of Mg-deficient leaves very significantly delayed expression of leaf chlorosis.

Production of quality seedling is of upmost important for wax gourd system and low seed germination rate has long been a challenge for wax gourd. Higher seed vigor and seedling establishment are closely correlated with better yield potential (Mondo et al., 2013). Reductions in the seed quality can be triggered by environmental stresses by which mother-plant are submitted (Carvalho et al., 2018). However, it was unknown whether Mg deficiency affects seeds and next-generation seedlings growth of wax gourd. The present study clearly showed that seeds from the low Mg mother plants had poor seed germination and abnormal seedling development (Figure 7), which were associated with lower amount of starch in seeds of Mg deficient plants (Table 5). Similar results were also reported in wheat plants grown under greenhouse conditions (Ceylan et al., 2016). Impairment in seed vigor and seedling establishment from low Mg wax gourd plants is probably related to reduced phloem transport and deposition of carbohydrates in the seeds under Mg deficiency. It is known that seeds with lower carbohydrate status have poor germination and seedling establishment capacity (Zhao et al., 2018). It is, therefore, of great importance to ensure a good Mg nutrition of the mother plants during the reproductive growth stage. In plants with particularly high sink size and demand for photoassimilates such as wax gourd plants, sufficiently high Mg concentrations need to be maintained during the late stage of growth to guaranty adequate carbon flow in the fruits.

## Conclusion

Magnesium deficiency significantly diminished fruit yield of wax gourd and yield potential of next generation on open-field, by limiting the carbohydrate transport from source organs to developing fruit. Ensuring sufficient Mg supply to plant species with high sink size such as wax gourd, during the reproductive growth stage, is a critical factor for achieving higher fruit yield formation and also better vigor of next-generation seeds. The results also showed that wax gourd could be an excellent model crop with its particular sink size for the Mg deficiency experiments.

## Data Availability Statement

All datasets generated for this study are included in the article/supplementary material.

## Author Contributions

YH and BZ designed the research. JF, CY, XC, and LW detected samples in the lab. DX participated in the experimental design. JC and ZS did field experiments. BZ and IC wrote the manuscript. IC revised the manuscript. All authors approved the final manuscript.

## Conflict of Interest

The authors declare that the research was conducted in the absence of any commercial or financial relationships that could be construed as a potential conflict of interest. The reviewer EP declared past co-authorship with one of the authors IC to the handling Editor.
